# Identification and Characterization of *Scutavirus testudinidalpha3* Infection in Captive Elongated Tortoises (*Indotestudo elongata*)

**DOI:** 10.1155/tbed/2984888

**Published:** 2026-08-11

**Authors:** Han-Yang Wang, Shih-Hsuan Liu, Wei-Hsiang Huang, Hui-Wen Chang, Chain-Ren Jeng, Chiu-Hung Cheng, Pao-Jung Wang, Yen-Chen Chang

**Affiliations:** ^1^ Graduate Institute of Molecular and Comparative Pathobiology, School of Veterinary Medicine, National Taiwan University, Taipei, Taiwan, ntu.edu.tw; ^2^ Taipei Zoo, Taipei, Taiwan

**Keywords:** glycoprotein B, *Indotestudo elongata*, in situ hybridization, *Scutavirus testudinidalpha3*

## Abstract

*Scutavirus testudinidalpha3*, formerly *Testudinid alphaherpesvirus* 3 or testudinid herpesvirus (TeHV)‐3, is a highly contagious herpesvirus that has been reported in multiple testudinid species, causing acute stomatitis–rhinitis complex in affected animals or establishing latent infections in surviving individuals. This study investigated an outbreak of *S. testudinidalpha3* infection in 17 elongated tortoises (*Indotestudo elongata*) housed exclusively in an outdoor zoo enclosure in Taiwan in March, 2022. The affected tortoises exhibited nasal and ocular discharge and diphtheroid plaques on the oral mucosa. Despite prompt isolation and supportive treatment, 14 individuals succumbed. Necropsy and histopathologic examination revealed severe necrosis with inflammation, characterized by eosinophilic intranuclear inclusion bodies and occasional syncytial cells in multiple organs. Conventional polymerase chain reaction (PCR) and in situ hybridization (ISH) confirmed *S. testudinidalpha3* infection. DNA polymerase (DNApol) gene sequences were identical among all individuals and shared 97.86% nucleotide similarity with the German 4295/7R strain. Furthermore, the full‐length sequence of glycoprotein B (gB), a potential virulence factor in herpesviruses, showed 99.9% identity with the Switzerland CH6883/03 strain, classifying this isolate as belonging to the genogroup B, known for its high pathogenicity. This report documents the first confirmed case of *S*. *testudinidalpha3* infection in elongated tortoises.

## 1. Introduction

Herpesvirus infections are significant global viral diseases documented across nearly all orders of reptiles, including chelonians [1]. Among these, testudinid herpesviruses (TeHVs) are a group of herpesviruses that infects numerous tortoise species, including those formerly designated as TeHV‐1, TeHV‐2, TeHV‐3, and TeHV‐4 [1–4]. However, only TeHV‐3 has been named as *Scutavirus testudinidalpha3*, belonging to the genus *Scutavirus* within the family Orthoherpesviridae, according to the current taxonomy by the International Committee on Taxonomy of Viruses (ICTV). *S. testudinidalpha3*, considered a Eurasian geographical subgroup, has been identified in a wide range of species, including Greek (*Testudo graeca*), Hermann’s (*T. hermanni*), Russian (*T. horsfieldii*), marginated (*T. marginata*), Egyptian (*T. kleinmanni*), African spurred (*Centrochelys sulcata*), and radiated tortoises (*Astrochelys radiata*) [5–8]. Another Eurasian subgroup, TeHV‐1, affects Russian, pancake (*Malacochersus tornieri*), Greek, Hermann’s, and leopard tortoises (*Stigmochelys pardalis*) [9–11]. In contrast, TeHV‐2 represents an American subgroup identified only in Agassiz’s desert tortoises (*Gopherus agassizii*) [12], while TeHV‐4 has been detected exclusively in African species, such as bowsprit (*Chersina angulata*) and leopard tortoises [3, 13]. Generally, infections caused by TeHV‐1 and *S. testudinidalpha3* result in severe clinical signs, specifically the ulcerative to diphtheroid‐necrotizing stomatitis–rhinitis complex, leading to high morbidity and mortality [1, 14]. Information regarding TeHV‐2 and TeHV‐4 remains limited. While TeHV‐2 has been associated with caseous oral plaques and death [12], TeHV‐4 infections are often asymptomatic [3] or present respiratory distress and hypersalivation [13]. While these four TeHVs contribute to varying clinical outcomes, species‐dependent susceptibility is also a major factor. Hermann’s tortoises are reported to be highly susceptible, often experiencing fatal diseases [2, 15], whereas Greek and Russian tortoises are typically less affected [16, 17]. It has also been proposed that species‐dependent susceptibility may be associated with the adaptation or coevolution of TeHVs within resistant species, thereby reducing lethality while still allowing transmission of the virus to more susceptible species or naïve individuals [1, 14].

Unlike the abovementioned tortoise species, herpesvirus infection in the elongated tortoise (*Indotestudo elongata*) is rarely reported. The elongated tortoise is a medium‐sized tortoise species distributed across Southeast Asia, including Thailand, India, Vietnam, Bangladesh, Nepal, Bhutan, Cambodia, Laos, and Malaysia [18–20]. Due to severe habitat loss, overexploitation, and forest fires, it is currently listed as a critically endangered species by the International Union for Conservation of Nature (IUCN) [21]. Numerous conservation programs now focus on habitat protection and population reinforcement [22], making the emergence of infectious diseases in this species a significant conservation concern.

Various diagnostic methods detect TeHV, including histopathology, virus isolation, electron microscopy, and molecular assays. While virus isolation is the definitive method [23], serological tests like enzyme‐linked immunosorbent assay (ELISA) and serum neutralization (SN) are essential for identifying latent infections in asymptomatic carriers. Detecting IgY antibodies against TeHV‐2 and *S. testudinidalpha3* have been developed, but only a limited number of tortoise species have been applied [12, 23, 24]. The SN test could be applied to detect TeHVs across tortoise species but is not widely available [8]. For symptomatic animals, polymerase chain reaction (PCR) provides a rapid diagnostic tool, and specific assay targets several genes that have already been described. A pan‐herpesvirus PCR assay capable of detecting all herpesviruses has been developed [25]. Additionally, specific PCR assays have been designed for particular TeHV subtypes, such as the DNA polymerase (DNApol) gene for TeHV‐1 [26], the helicase/primase complex (HPC) for *S. testudinidalpha3* [27], and the ribonucleotide reductase large subunit for TeHV‐2 and *S. testudinidalpha3* [8, 12, 28].

Recently, glycoprotein B (gB), a surface protein of *S. testudinidalpha3*, has gained attention because it is highly conserved among herpesviruses and exhibits sequence variability correlated with antigenicity and immunogenicity in other species [29, 30]. gB plays a critical role in mediating viral fusion and entry as well as in cell‐to‐cell spread, making it a promising target for therapeutic and vaccine development [31, 32]. Moreover, gB has been implicated as a potential virulence factor in cytomegalovirus and herpes simplex virus (HSV) 1 [33, 34]. Origgi et al. [35] demonstrated that *S. testudinidalpha3* can be divided into two distinct genogroups (A and B) and one recombinant genogroup (C) based on amino acid substitutions at specific positions, involving a total of 66 single nucleotide polymorphisms (SNPs). Of these, 33 SNPs uniquely differentiate genogroup A from B, but only five SNPs are missense and all located at the 3′ half of the gene. These substitutions include asparagine (N) to lysine (K) at position 507, asparagine (N) to aspartate (D) at position 520, and valine (V) to isoleucine (I) at position 615. In genogroup C, the amino acids at these three positions are, respectively, K, D, and V, representing an intermediate profile between genogroups A and B. Clinically, infections with genogroup B viruses are associated with greater severity, characterized by inclusion bodies in multiple tissues and a higher incidence of pneumonia and meningitis, whereas genogroup A infections tend to be localized, with no inclusion bodies and rare pneumonia [35].

In this report, we describe the first confirmed outbreak of *S. testudinidalpha3* infection in elongated tortoises. We conducted a comprehensive investigation, including necropsy, histopathology, molecular diagnostics, and phylogenetic analyses. Furthermore, in situ hybridization (ISH) was developed and demonstrated to be a valuable diagnostic tool for identifying diseases in exotic species, which are often difficult to study and poorly understood.

## 2. Materials and Methods

### 2.1. Animals

Seventeen elongated tortoises were housed exclusively in an outdoor exhibition at a local zoo in Taiwan. The enclosure had maintained a closed population for over 10 years, with no new individuals introduced during this period. These tortoises, confiscated from the illegal wildlife trade, were kept at the zoo for conservation purposes. The animals ranged in age from 8 to 35 years. This group consisted of five males, nine females, and three of undetermined sex. In early March 2022, 10 individuals presented with anorexia and respiratory signs accompanied by oral, nasal, and ocular discharges; the remaining seven tortoises became symptomatic over the following two to 6 days. A caseous material was observed in the oral cavities of several individuals. Symptomatic tortoises were immediately isolated and provided with supportive treatments, including antibiotics (enrofloxacin and metronidazole), lactated Ringer solution, and an antiviral drug (acyclovir 80 mg/kg). Despite these interventions, 14 individuals died ultimately, while only three survived. The median time from onset of clinical signs to death of these dead animals was 15 days, with a maximum survival time of 37 days.

### 2.2. Gross and Histopathological Examination

All the died tortoises were necropsied and sampled for further analyses. During necropsy, gross lesions were documented, and representative tissues, including the heart, liver, spleen, lung, kidney, gastrointestinal tract, reproductive tract, and central nervous system (CNS), were fixed in 10% neutral‐buffered formalin. Fresh tissues showing prominent gross lesions (such as oral mucosa, tongue, esophagus, or lung) were collected and stored at −20°C for subsequent DNA extraction. Formalin‐fixed paraffin‐embedded (FFPE) tissue blocks were prepared according to standard protocols, sectioned at 4 µm, and stained with hematoxylin and eosin (H&E) for histopathological examination.

### 2.3. DNA Extraction From Fresh Tissue or Oral Swabs

Total DNA from each case was extracted from fresh tissues showing severe lesions in dead tortoises or oral swabs collected from three surviving tortoises in the same exhibition using the DNeasy Blood & Tissue Kit (Qiagen, Valencia, CA, USA) following the manufacturer’s instructions. The extracted DNA was stored at −20°C for PCR assays.

### 2.4. PCR and Nucleotide Sequencing

Before performing PCR for detecting the TeHV, the tortoise β‐actin gene was used as an internal control to ensure the quality of extracted DNA samples. Subsequently, a nested PCR assay was conducted to amplify a partial sequence of the DNApol gene to detect a broad range of herpesviruses. A *S. testudinidalpha3*‐specific PCR targeting the DNA HPC (UL5 homolog) was then employed to confirm the specific TeHV species. Finally, the full‐length gB gene, which potentially contributes to viral virulence, was amplified. All PCR assays were performed using amaR OnePCR (GeneDireX Inc., Taipei, Taiwan). Primers and conditions are summarized in Table 1. β‐Actin was amplified using primers F2 and U2 under the following conditions: 94°C for 1 min; 35 cycles of 94°C for 30 s, 46°C for 30 s, and 72°C for 30 s; and a final extension at 72°C for 8 min, yielding a 238‐bp amplicon [8]. For the partial DNApol gene [25], nested PCR was performed as follows: initial denaturation at 94°C for 3 min, followed by 35 cycles of 94°C for 30 s, 46°C for 60 s, and 72°C for 60 s, and a final extension at 72°C for 7 min. The first round used two forward primers (DFA and ILK) and one reverse primer (KG1); the second round included 2 µL of the first‐round product with the primer pair TGV/IYG under the same parameters, yielding a 215–235 bp amplicon. PCR targeting the HPC used primers UL5‐F and UL5‐R [27], yielding a 307 bp amplicon. The cycling conditions were 95 °C for 3 min, 35 cycles of 95°C for 30 s, 55°C for 30 s, and 72°C for 30 s, and a final extension at 72°C for 10 min. For the full‐length gB gene (2484 bp), primers gBTeHV3FW and gBTeHV3RV were used [35]. The PCR program comprised an initial denaturation at 95°C for 1 min; 40 cycles of 95°C for 30 s, 50°C for 30 s, and 68 °C for 1 min; and a final elongation at 68°C for 10 min.

**Table 1:** Primer sets applied for *Scutavirus testudinidalpha3* in this study.

PCR assay	Target gene	Primer name	Sequence	Reference
Pan‐herpesvirus	DNA polymerase	DFA	5′—GAYTTYGCNAGYYTNTAYCC—3′	[21]
		ILK	5′—TCCTGGACAAGCAGCARNYSGCNMTNAA—3′	
		KG1	5′—GTCTTGCTCACC AGNTCNACNCCYTT—3′	
		TGV	5′—TGTAACTCGGTGTAYGGNTTYACNGGNGT—3′	
		IYG	5′—CACAGAGTCCGTRTCNCCRTADAT—3′	
*Scutavirus testudinidalpha3*	Helicase/primase complex	UL5‐F	5′—TGGTTGGACAACAACATGGC—3′	[23]
		UL5‐R	5′—TCATTCCGTTATACATGAGCG—3′	
Internal control	β‐Actin	F2	5′—TGCTTCTAAACCGGACTG—3′	[6]
		R2	5′—AAATGTACAATCAAAGTCC—3′	
Full‐length gB	gB	gBTeHV3FW	5′—AAAATGATCATGTGGTTATCGTT—3′	[31]
		gBTeHV3RV	5′—AAAATTATTGGGAGGAATCGTCTATCT—3′	
		gB‐F1	5′—ACCTATGTGGAATTCACCTGGGTAATAGCG—3′	
		gB‐F2	5′—ACCGGGGATGTGGTCGAGATGTCTCCATTC—3′	
		gB‐F3	5′—GGTACAAGGATACTCATCTGCGAGTTAACG—3′	
		gB‐F4	5′—TCAATCTCGACCTCACGATGCTGGAGAACG—3′	
		gB‐R	5′—TTTATGACACGGGCTAGGCGGAGATAGGCG—3′	

All PCR products were separated by electrophoresis on 2% agarose gels containing ethidium bromide at 110 V for 30 min. Bands of expected sizes were visualized using a Gel Doc XR imaging system (BioRad, Hercules, CA, USA), excised, purified with the QIAquick Gel Extraction Kit (Qiagen), and submitted for Sanger sequencing (Tri‐IBiotech Inc.). Nucleotide sequences were confirmed by using the Basic Local Alignment Search Tool (BLAST) against the databases of the National Center for Biotechnology Information (NCBI). Sequencing primers for the gB gene are listed in Table 1.

### 2.5. Sequence and Phylogenetic Analysis

Alignments of DNApol, HPC, and full‐length gB sequences were conducted by using MEGA X software [36]. Phylogenetic trees were constructed in all analyzed genes using the maximum likelihood method combined with the Kimura 2‐parameter model with bootstrapping for 1000 replications. MegAlign of DNAstar Bioinformatics Software (DNAstar, Madison, WI) was used to compare the sequence identity among various TeHV strains.

### 2.6. Plasmid Construction and Cloning

Purified PCR products of the partial DNApol gene and HPC gene were ligated into the pCR2.1 vector (Thermo Fisher Scientific Inc., Waltham, MA, USA) using the TA Cloning Kit (Thermo Fisher Scientific Inc.) according to the manufacturer’s protocol, followed by transformation into *E. coli* One Shot TOP10 chemically competent cells. Six colonies were randomly selected and cultured in 3 mL of LB broth supplemented with 50 µg/mL kanamycin at 37°C, 120 rpm overnight (16–18 h). Plasmids were extracted with the QIAprep Spin Miniprep Kit (Qiagen) and sequenced. The resulting sequences were compared with NCBI databases. Plasmids were stored at −20°C, and the corresponding LB cultures were mixed with 10% glycerol and preserved at −80°C.

### 2.7. ISH

The representative tissues of the severely affected organs were used for ISH, including the oral cavity, nasal cavity, esophagus, trachea, lung, liver, kidney, and pancreas. The 4 mm thick FFPE tissue slices on the coated slides were heated at 60°C for 30 min and subsequently deparaffinized and rehydrated by routine protocols. The tissue sections underwent different proteolytic digestion methods for antigen retrieval. The slices stained with the DNApol gene probe were interacted with 50 mM tris‐CaCl_2_ buffer containing 20 mg proteinase K at 37°C for 1 h; as for the HPC probe, the slices were applied for 50 mM tris‐CaCl_2_ buffer supplemented with proteinase K (1:20 dilution) for 30 min and then 20 mg/mL proteinase K in phosphate‐buffered saline for 30 min. The antigen retrieval was ended by adding 0.4 % formalin. Following, the slides were washed with TBST buffer (Genestar Biotechnology, Kaohsiung, Taiwan) three times. Each DNA digoxigenin‐labeled probe, which was synthesized by using PCR DIG Probe Synthesis Kit (Roche Diagnostics, Mannheim, Germany) with the constructed plasmid and then diluted 40 times with Hybridization Buffer of the PCR/DIG ELISA system (Roche Diagnostics), was added onto the tissue slices. The slides were first sealed by a siliconized coverslip and then heated for 6 min at 95°C, followed by a cold shock for 1 min. Next, these tissue slices were incubated overnight at 42 and 47°C, respectively, on an IQ Kinetic Slide Stainer (Biocare Medical, Concord, CA, USA). After the hybridization, stringent washes were conducted by using SSC buffer at room temperature (RT) for 15 min and then applying 20 times diluted SSC buffer at 42°C for 15 min. The tissue slices were first interacted with 3% H_2_O_2_ for 12 min at RT and then blocked with 10% Goat Serum (Dako, Cambridgeshire, UK) at RT for 1 h. After removing the blocking solution, anti‐digoxigenin (Roche Diagnostics) in 500 dilutions with 10% goat serum (Dako) was directly added onto the tissue at RT for 1 h incubation. Finally, the tissue slices were developed with the EnVision+ Dual Link System‐HRP (3,3‐diaminobenzidine) (Dako) according to the manufacturer’s protocol and were counterstained with hematoxylin. The result was evaluated by using an optical microscope. The positivity was interpreted as brown signals within the epithelial cells or intranuclear inclusion bodies.

## 3. Results

### 3.1. Gross and Histopathological Lesions

In appearance, the affected tortoises had severe subcutaneous edema (9/14), and some of them showed redness at bilateral nostrils and eyelids (4/14), occasionally with small amounts of caseous nasal discharge (2/14). After removing the plastron, small to moderate amounts of yellowish transparent effusion were accumulated in the celom (2/14). The oral mucosa and tongue were severely reddened (2/14) and usually covered with abundant caseous material admixed with some mucus (11/14). The mucosa of the esophagus was reddened and covered with variable amounts of similar caseous material as described in the oral cavity (8/14), which sometimes extended to the cardiac mucosa in some individuals (4/14). The small intestinal mucosa at the proximal end was slightly reddened (3/14) or covered with some caseous material (1/14). Similar lesions were also observed in the mucosa of the respiratory tract, including the trachea (7/14), bilateral bronchi (10/14) and the adjacent pulmonary parenchyma (4/14). Diffuse serous atrophy of the adipose tissue was observed in the subcutis and visceral fat (5/14). The liver was mildly swollen and yellow (10/14).

Microscopically, there were multifocal to coalescing mucosal ulcerations with accumulation of large amounts of necrotic cell debris intermingled with fibrin and occasional colonies of gram‐positive rods in the nasal and oral cavities, tongue, pharynges, esophagus, trachea, lungs, stomach, and proximal small intestine. Infiltration of moderate and large numbers of inflammatory cells, such as heterophils, macrophages, lymphocytes, and plasma cells, was observed in the affected mucosa and the underlying lamina propria with varying degrees of edema. Additionally, there were variable numbers of eosinophilic intranuclear inclusion bodies (mostly Cowdry type A and occasionally type B) in the syncytial cells, the affected lining epithelium, and the adjacent glandular epithelium. Occasionally, the intranuclear inclusion bodies were also observed in the liver (2/14), pancreas (1/14) and kidney (1/14) accompanied by prominent inflammation. Other lesions such as hepatocellular vacuolar degeneration (8/14) and nematoda infestation in the intestinal tract (5/14) were noted. The representative gross and microscopic findings are illustrated in Figures 1 and 2.

**Figure 1:**
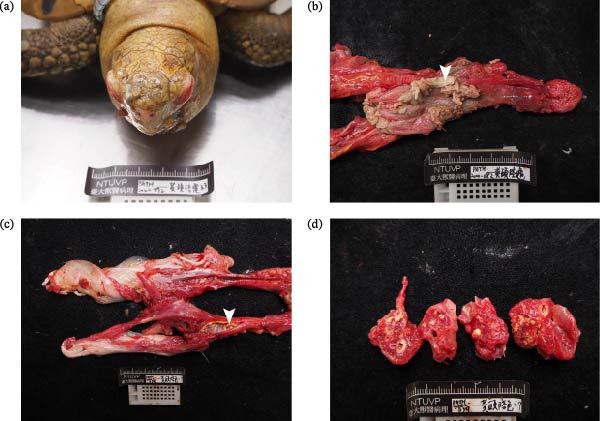
Gross lesions and histopathological findings in elongated tortoises infected with *Scutavirus testudinidalpha3*. The tortoises showed severe subcutaneous edema with erythema of the eyelids and nostrils (a). Yellowish, caseous pseudomembrane (arrowheads) was present in the oral cavity and esophagus (b), trachea (c), and sectioned lung (d).

**Figure 2:**
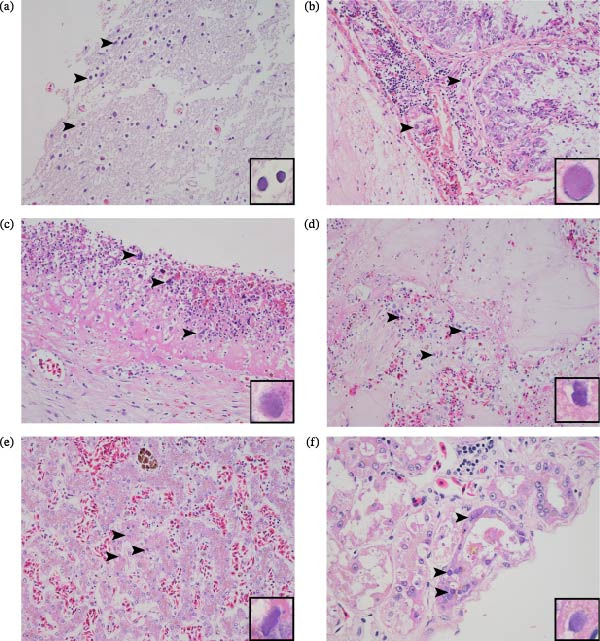
Histopathological findings in elongated tortoises infected with *Scutavirus testudinidalpha3*. Eosinophilic intranuclear inclusion bodies (arrowheads) were detected in the severely affected organs including brain (a), nasal cavity (b), trachea (c), lung (d), liver (e), and kidney (f). Images are shown at 200x magnification with hematoxylin and eosin (H&E) staining; insets show higher magnification at 1000x.

### 3.2. Detection of TeHV via PCR

DNA was successfully extracted from representative tissues and oral swabs of all 14 dead tortoises and the three survivors, as confirmed by PCR targeting the tortoise β‐actin gene. All cases presented target amplicons for both DNApol and HPC via gel electrophoresis, indicating *S. testudinidalpha3* infection. The gB gene was also successfully detected in all cases.

### 3.3. Sequence Analysis

Sequenced genes, including the partial DNApol, HPC, and three full‐length gB, were deposited in GeneBank under the accession numbers OR050596‐OR050609, OR050610‐OR050623, and OQ973472–OQ973474, respectively. Based on Blast analysis of the 14 partial DNApol sequences, the isolates were most similar to *S. testudinidalpha3*, which was the most common and virulent strain in tortoises. These isolates shared 96.67%–97.86% sequence identity with other published isolates, including accession numbers DQ343883 and AB047545 for TeHV‐1, AY916792 for TeHV‐2, KM924293.1, KM924292 for *S. testudinidalpha3*, and GQ222415 for TeHV‐4. The most closely related sequence was the German 4295/7R strain (Accession Number KM924293.1), identified in 1995 from a Hermann’s tortoise. To further confirm the viral species, a *S. testudinidalpha3*‐specific PCR assay targeting the HPC was conducted, and the results showed 100% sequence identity with published strains.

The full‐length gB sequences of all animals in the present study shared 100% nucleotide identity but 98.2%–99.9% nucleotide identity and 99.5% to 100% amino acid identity with the published strains. The most identical sequence was strain CH6883/03 (KP979730), isolated from a Hermann’s tortoise in Switzerland in 2003. To investigate potential virulence factors, key nucleotide sequences and amino acid residues were examined [35]. Comparison with published partial gB sequences (nt 1420–2478) showed 95.7%–100% nucleotide identity, while the partial protein region (aa 474–826) showed 98.9%–100% identity (Figure 3). Based on the gB sequence, all isolates in this study were classified as genogroup B, characterized by three specific amino acid substitutions at positions 507, 520, and 615 compared to those in genogroup A (Table 2).

**Figure 3:**
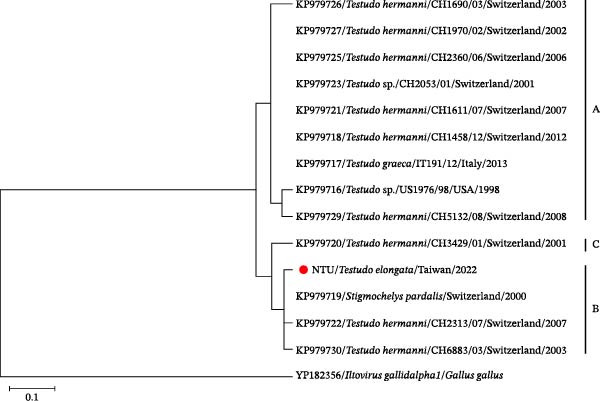
Comparison of the partial amino acid sequence of glycoprotein B (position 474–826) among *Scutavirus testudinidalpha3* strains. Genogroups A, B, and C are classified based on amino acid variations at positions 507, 520, and 615. • Indicates the isolate in the present study.

**Table 2:** Comparison of amino acid changes between our isolates and other published strains.

Data obtained	Strain	Accession number	Genogroup	Amino acid position		
				507	520	615
Reference strain	US1976/98	KP979716	A	N (asparagine)	N (asparagine)	V (valine)
	IT191/12	KP979717	A	N (asparagine)	N (asparagine)	V (valine)
	CH1458/12	KP979718	A	N (asparagine)	N (asparagine)	V (valine)
Reference strain	CH7204/00	KP979719	B	K (lysine)	D (aspartate)	I (isoleucine)
	CH2313/07	KP979722	B	K (lysine)	D (aspartate)	I (isoleucine)
	CH6883/03	KP979730	B	K (lysine)	D (aspartate)	I (isoleucine)
Current study	NTU/716/2023	OQ973472	B	K (lysine)	D (aspartate)	I (isoleucine)
	NTU/791/2023	OQ973474	B	K (lysine)	D (aspartate)	I (isoleucine)
	NTU/697/2023	OQ973474	B	K (lysine)	D (aspartate)	I (isoleucine)
Reference strain	CH3429/01	KP979720	C	K (lysine)	D (aspartate)	V (valine)

### 3.4. ISH

To correlate viral DNA with histopathological lesions, ISH targeting the DNApol and HPC genes of *S. testudinidalpha3* was performed in all 14 PCR‐positive cases. Positive hybridization signals were detected within intranuclear inclusion bodies in all affected organs, including the oral and nasal cavities, tongue, trachea, bronchi, lungs, esophagus, stomach, and proximal small intestine. Approximately 50%–60% of the inclusion bodies showed positive ISH signals. Representative ISH results using different probes in various tissues are shown in Figure 4.

**Figure 4:**
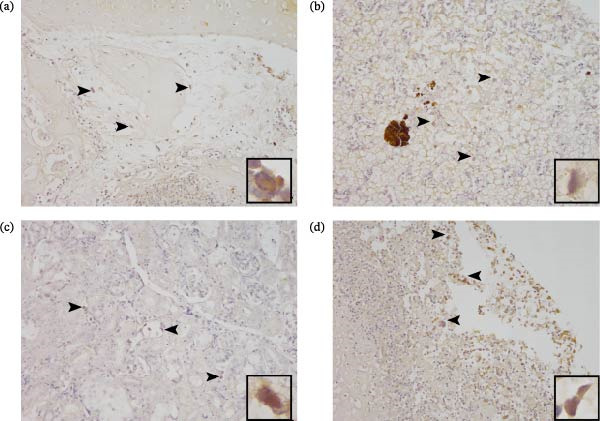
Detection of the DNA polymerase and helicase/primase complex genes of *Scutavirus testudinidalpha3* in formalin‐fixed, paraffin‐embedded tissues by in situ hybridization. Positive hybridization signals (brown) are visible within intranuclear inclusion bodies (arrowheads) in multiple organs, including the lung (a), liver (b), kidney (c), and trachea (d). Images are shown at 200x magnification; insets show higher magnification at 1000x.

## 4. Discussion

In this study, we report the first detection of *S. testudinidalpha3* infection in elongated tortoises (*I. elongata*). To confirm the infection, a comprehensive investigation was conducted, including necropsy, histopathological examination, molecular diagnosis, ISH, and phylogenetic analysis of the outbreak involving 17 elongated tortoises housed in a local zoo in Taiwan. The histopathological lesions observed in 14 deceased individuals and the tissue tropism characterized by ISH were consistent with those previously described in other tortoise species infected with *S. testudinidalpha3*. Furthermore, based on the amino acid sequence analysis of gB and histopathological findings, the isolates from this outbreak were classified as genogroup B.

Gross lesions in the affected tortoises, characterized by the stomatitis–rhinitis complex, were compatible with previous reports [5, 11, 37, 38]. The oral and respiratory mucosa were covered with variable amounts of caseous pseudomembrane. Microscopically, intranuclear inclusion bodies have been detected not only in the epithelium of the kidney, stomach, and intestine but also in the nuclei of submucosal and myenteric ganglion cells in Russian tortoises [27]. Similarly, in the present study, inclusion bodies were observed in the epithelium of the respiratory and upper digestive tracts, hepatocytes, the renal tubular epithelium, pancreatic exocrine glands, and neurons of the cerebellum and cerebrum. Although hepatitis with inclusion bodies has been described in *T. horsfieldii*, hepatic necrosis is seldom reported in *S. testudinidalpha3* infection [39]; however, similar findings were identified in two tortoises in this outbreak. As in human and psittacine herpesviruses, TeHV appears to have a broad range of target cells, likely due to the widespread distribution of cellular receptors [40, 41]. For instance, heparan sulfate proteoglycans (HSPGs) are known to mediate herpesvirus attachment and entry, and tissues with high HSPG expression are often the most severely affected [42, 43]. Nonetheless, TeHV infection is also influenced by host factors such as species, age, and individual susceptibility [35].

Using a pan‐herpesvirus screening PCR targeting the partial DNApol gene and a confirmatory *S. testudinidalpha3*‐specific PCR assay (HPC), we identify the etiologic agent of this outbreak as *S. testudinidalpha3*. Compared to other published isolates, the partial DNApol gene sequence in our strains shared 97.86% nucleotide similarity with the German 4295/7R strain. However, the pan‐herpesvirus screening PCR targets only a partial segment of the DNApol gene, located at the relatively conserved area and providing limited phylogenetic resolution. Regarding HPC gene detection, the primer pair used in the present study has been published; however, no corresponding sequences have been deposited in NCBI databases to date. In contrast, although the gB gene analyzed in this study was sequenced in full length, most deposited sequences are partial rather than full length. Therefore, the nucleotide region spanning positions 474–826 was selected as it allows inclusion of the largest number of available sequences for subsequent phylogenetic analysis. Although full‐genome comparisons of *S. testudinidalpha3* would provide more comprehensive insights into viral evolution and phylogeny, such analyses remain challenging due to the limited availability of complete genome sequences and technical constraints associated with whole‐genome sequencing. Next‐generation sequencing (NGS) is a powerful approach for obtaining full‐length viral genome sequences, particularly for herpesviruses, which possess large genomes. However, this technique has several limitations, including the need for successful viral isolation and propagation to obtain sufficient viral material or the availability of a host reference genome to enable exclusion of background host sequences and accurate assembly of viral reads. In the present study, viral isolation was unsuccessful, and no tortoise reference genome is currently available in public databases; therefore, a complete viral genome sequence could not be generated.

*S. testudinidalpha3* has been frequently detected in pet tortoises and is associated with high morbidity and mortality in certain species, such as the Hermann’s tortoise. Before this outbreak, reported TeHV infections have primarily occurred within the genera *Testudo*, *Centrochelys*, and *Astrochelys* [1, 14]. Previous large‐scale epidemiological studies in Europe demonstrated TeHV detection rates of 6.5% (321/4797) and 8.0% (81/1015), with approximately half of all positive cases identified as *S. testudinidalpha3* (49.0% and 54.3%, respectively) [17, 44]. Notably, these surveys found no evidence of TeHV infection in elongated tortoises. In contrast, the current outbreak confirms that elongated tortoises are not only susceptible to *S. testudinidalpha3* but also exhibit a high mortality rate (82.4%, 14/17), similar to that of Hermann’s tortoises [11], despite medical intervention. Conversely, Greek tortoises have been reported to possess higher relative resistance to TeHV infection [45]. Beyond species‐specific resistance, gB has been identified as a potential virulence factor in *S. testudinidalpha3*. Specifically, viral strains belonging to genogroup B, characterized by specific amino acid substitutions at positions 507, 520, and 615 of the gB protein, are associated with a higher frequency of intranuclear inclusion bodies, multiorgan involvement, pneumonia, and CNS lesions in infected tortoises [35]. Although only three isolates were previously characterized in that study, our findings align closely with those observations, suggesting that these key amino acid residues may correlate with virulence. Further investigation is warranted.

Compared with the limited number of full‐length gB sequences available in GenBank, strain CH6883/03 (Accession Number KP979730), isolated from a Hermann’s tortoise in Switzerland in 2003, shared the highest sequence identity (99.9%) with our isolates. The affected tortoises in this study had been housed in the same outdoor enclosure for over 10 years without new introductions, and all isolates were genetically identical and closely related to strains detected nearly two decades ago. Following the onset of clinical signs, all zookeepers and equipment that had contact with the enclosure were subjected to PCR testing to investigate potential transmission routes, and all results were negative. These findings suggest that the outbreak may have originated within the enclosure through reactivation of a latent infection in one or more tortoises, followed by horizontal transmission within the group, rather than by the recent introduction of a new virus [35]. However, the precise timing of viral introduction into the population could not be determined.

Given the potential for severe disease in susceptible tortoises, minimizing risk factors for herpesvirus infection is essential. The most critical factor is the presence of latent carriers within a collection [6]. Reactivation of latent TeHV infection is often triggered by stressors that suppress host immunity, enabling viral replication and re‐entry into the lytic phase [1]. The posthibernation period may also contribute as temperatures between March and April are near the optimal range for TeHV replication [6]. Immunosuppression is a well‐known trigger for herpesvirus reactivation [46]. In humans, HSV reactivation can be provoked by psychological stress, fatigue, and exposure to bright light [47], while in mice, elevated body temperature has been shown to induce HSV activation [48]. In tortoises, suboptimal environmental temperature below the preferred optimal temperature zone (26–29°C) can suppress immune function, predisposing animals to infections such as *Mycoplasma* [49]. Notably, a sharp temperature drop occurred in late February 2022, which may have acted as a significant stressor precipitating the disease onset in this outbreak.

Tortoises infected with TeHV should therefore be considered lifelong carriers and should be permanently segregated from uninfected individuals [1]. Furthermore, Marenzoni et al. [6] noted that even long‐term isolation may not fully prevent *S. testudinidalpha3* outbreaks. In this outbreak, the disease course lasted approximately two to 3 weeks, consistent with previous reports [1]. In the end, three tortoises survived, but one died without detectable virus and TeHV‐associated lesions 2 months later. Because survived individuals may harbor latent infection and serve as potential carriers, they must be managed with strict biosecurity measures. Avoiding new animal introductions, maintaining rigorous environmental hygiene, and preventing stressors such as overcrowding and cold exposure are critical to reducing the risk of TeHV reactivation and transmission. To date, two surviving tortoises were periodically monitored by PCR with oral swabs and showed no clinical signs. They were continuously quarantined in the original exhibition, and cleaning equipment was disinfected and separated from the other groups to avoid cross‐contamination. For consistently monitoring *S. testudinidalpha3* infection in the survived animals and early disease diagnosis in other tortoises, a standard PCR method has been established in the laboratory of the zoo. To prevent new outbreaks, individuals rescued from the wild or illegal trades and showing similar clinical signs will be tested immediately, and PCR‐positive animals will be kept isolated and administered with supportive treatments. In addition, tissue samples from dead tortoises showing similar lesions, such as ulcers in the gastrointestinal tract, will be routinely submitted for TeHV PCR for disease control. Until December 2025, no diseased tortoises or tissue samples collected from dead tortoises, except for rescued individuals, tested positive for TeHV.

## 5. Conclusion

This study presents the first report of *S. testudinidalpha3* infection in elongated tortoises, confirmed through histopathological, molecular, and ISH analyses. Sequence analysis revealed that the isolates were identical and shared high sequence similarity with strains previously detected in Europe in the early 2000 s. The outbreak was likely caused by viral reactivation in one or more tortoises with latent infection, followed by transmission among individuals within the same enclosure. Our findings underscore the importance of regular pathogen surveillance, strict biosecurity measures, and environmental management to minimize the risk of herpesvirus transmission and reactivation in zoological facilities.

## Funding

This research received no specific grant from any funding agency in the public, commercial, or not‐for‐profit sectors.

## Ethics Statement

All samples used in this study were collected as part of routine clinical diagnosis or standard postmortem examinations for disease investigation. No animals were euthanized or subjected to invasive procedures solely for the purpose of this research. Consequently, formal ethical approval was waived by the Institutional Animal Care and Use Committee (IACUC). All procedures complied with standard veterinary diagnostic and ethical guidelines.

## Conflicts of Interest

The authors declare no conflicts of interest.

## Data Availability

The sequences are available in GenBank: partial herpesvirus DNA polymerase (OR050596–OR050609), testudinid herpesvirus helicase/primase complex (UL5) (OR050610–OR050623), and full‐length gB gene (OQ973472–OQ973474).

## References

[1] Origgi F. C. , Testudinid Herpesviruses: A Review, Journal of Herpetological Medicine and Surgery. (2012) 22, no. 1-2, 42–54, 10.5818/1529-9651-22.1-2.42.

[2] Marschang R. E. , Viruses Infecting Reptiles, Viruses. (2011) 3, no. 11, 2087–2126, 10.3390/v3112087. 22163336 PMC3230843

[3] Bicknese E. J. , Childress A. L. , and Wellehan J. F. X.Jr, A Novel Herpesvirus of the Proposed Genus Chelonivirus From an Asymptomatic Bowsprit Tortoise (*Chersina angulata*), Journal of Zoo and Wildlife Medicine. (2010) 41, no. 2, 353–358, 10.1638/2009-0214R.1. 20597234

[4] Gandar F. , Wilkie G. S. , and Gatherer D. , et al.The Genome of a Tortoise Herpesvirus (Testudinid Herpesvirus 3) Has a Novel Structure and Contains a Large Region That Is Not Required for Replication *In Vitro* or Virulence *In Vivo* , Journal of Virology. (2015) 89, no. 22, 11438–11456, 10.1128/JVI.01794-15. 26339050 PMC4645648

[5] Heldstab A. and Bestetti G. , Herpesviridae Causing Glossitis and Meningoencephalitis in Land Tortoises (*Testudo hermanni*), Herpetopathologia. (1989) 1, no. 2, 5–9.

[6] Marenzoni M. L. , Santoni L. , and Felici A. , et al.Clinical, Virological and Epidemiological Characterization of an Outbreak of Testudinid Herpesvirus 3 in a Chelonian Captive Breeding Facility: Lessons Learned and First Evidence of TeHV3 Vertical Transmission, PLoS ONE. (2018) 13, no. 5, 10.1371/journal.pone.0197169, e0197169. 29746560 PMC5944942

[7] Marschang R. E. , Reptilien Z. , Papp T. , Ferretti L. , Hochscheid S. , and Bentivegna F. , Detection and Partial Characterization of Herpesviruses From Egyptian Tortoises (*Testudo kleinmanni*) Imported Into Italy From Libya, Journal of Zoo and Wildlife Medicine. (2009) 40, no. 1, 211–213, 10.1638/2008-0045.1. 19368266

[8] Origgi F. , Romero C. , and Bloom D. , et al.Experimental Transmission of a Herpesvirus in Greek Tortoises (*Testudo graeca*), Veterinary Pathology. (2004) 41, no. 1, 50–61, 10.1354/vp.41-1-50. 14715968

[9] Stöhr A. C. and Marschang R. E. , Detection of a Tortoise Herpesvirus type 1 in a Hermann’s Tortoise (*Testudo hermanni* Boettgeri) in Germany, Journal of Herpetological Medicine and Surgery. (2010) 20, no. 2-3, 61–63, 10.5818/1529-9651-20.2.61.

[10] Une Y. , Murakami M. , Uemura K. , Fujitani H. , Ishibashi T. , and Nomura Y. , Polymerase Chain Reaction (PCR) for the Detection of Herpesvirus in Tortoises, Journal of Veterinary Medical Science. (2000) 62, no. 8, 905–907, 10.1292/jvms.62.905. 10993191

[11] Une Y. , Uemura K. , Nakano Y. , Kamiie J. , Ishibashi T. , and Nomura Y. , Herpesvirus Infection in Tortoises (*Malacochersus tornieri* and *Testudo horsfieldii*), Veterinary Pathology. (1999) 36, no. 6, 624–627, 10.1354/vp.36-6-624. 10568449

[12] Johnson A. J. , Pessier A. P. , Wellehan J. F. , Brown R. , and Jacobson E. R. , Identification of a Novel Herpesvirus From a California Desert Tortoise (*Gopherus agassizii*), Veterinary Microbiology. (2005) 111, no. 1-2, 107–116, 10.1016/j.vetmic.2005.09.008. 16253444

[13] Kolesnik E. , Mittenzwei F. , and Marschang R. E. , Detection of Testudinid Herpesvirus type 4 in a Leopard Tortoise (*Stigmochelys pardalis*), Tierärztliche Praxis Ausgabe K: Kleintiere/Heimtiere. (2017) 44, no. 4, 283–286, 10.15654/TPK-150843. 27301060

[14] Okoh G. R. , Horwood P. F. , Whitmore D. , and Ariel E. , Herpesviruses in Reptiles, Frontiers in Veterinary Science. (2021) 8, 10.3389/fvets.2021.642894, 642894. 34026888 PMC8131531

[15] Marenzoni M. L. , Santoni L. , Rossi E. , and Origgi F. C. , Long-Term Monitoring of Four Scutavirus Testudinidalpha3-Infected Mediterranean Tortoises (Testudo Spp.) From a 2013 Outbreak: A Case Study, Veterinaria Italiana. (2025) 61, no. 1, 10.12834/VetIt.3533.26977.2. 40012404

[16] Hunt C. J.-G. , Herpesvirus Outbreak in a Group of Mediterranean Tortoises (Testudo Spp), Veterinary Clinics of North America: Exotic Animal Practice. (2006) 9, no. 3, 569–574, 10.1016/j.cvex.2006.05.018. 16931376

[17] Leineweber C. , Müller E. , and Marschang R. E. , Herpesviruses in Captive Chelonians in Europe Between 2016 and 2020, Frontiers in Veterinary Science. (2021) 8, 1195. 10.3389/fvets.2021.733299 PMC8549816 34722701

[18] Ihlow F. , Geissler P. , Sovath S. , Handschuh M. , and Böhme W. , Observations on the Feeding Ecology of *Indotestudo elongata* (Blyth, 1853) in the Wild in Cambodia and Vietnam, Herpetol Notes. (2012) 5, 5–7.

[19] Khan S. , Nath A. , and Das A. , The Distribution of the Elongated Tortoise (*Indotestudo elongata*) on the Indian Subcontinent: Implications for Conservation and Management, Herpetological Conservation and Biology. (2020) 15, no. 1, 212–227.

[20] Sriprateep K. , Aranyavalai V. , Aowphol A. , and Thirakhupt K. , Population Structure and Reproduction of the Elongated Tortoise *Indotestudo elongata* (Blyth, 1853) at Ban Kok Village, Northeastern Thailand, Tropical Natural History. (2013) 13, no. 1, 21–37, 10.58837/tnh.13.1.103039.

[21] Rahman S. , Platt K. , and Das I. , et al. *Indotestudo elongata* (Errata Version Published in 2019). The IUCN Red List of Threatened Species, 2019.

[22] Ihlow F. , Spitzweg C. , and Flecks M. , et al.Unexpected Lack of Genetic and Morphological Divergence in a Widespread Tortoise—Phylogeography of *Indotestudo elongata* , Salamandra. (2024) 60, no. 3.

[23] Origgi F. , Klein P. , and Mathes K. , et al.Enzyme-Linked Immunosorbent Assay for Detecting Herpesvirus Exposure in Mediterranean Tortoises (spur-Thighed Tortoise [*Testudo graeca*] and Hermann’s Tortoise [*Testudo hermanni*]), Journal of Clinical Microbiology. (2001) 39, no. 9, 3156–3163, 10.1128/JCM.39.9.3156-3163.2001. 11526144 PMC88312

[24] Jacobson E. R. , Berry K. H. , and Wellehan J. F.Jr, et al.Serologic and Molecular Evidence for Testudinid Herpesvirus 2 Infection in Wild Agassiz’s Desert Tortoises, *Gopherus agassizii* , Journal of Wildlife Diseases. (2012) 48, no. 3, 747–757, 10.7589/0090-3558-48.3.747. 22740541

[25] VanDevanter D. R. , Warrener P. , and Bennett L. , et al.Detection and Analysis of Diverse Herpesviral Species by Consensus Primer PCR, Journal of Clinical Microbiology. (1996) 34, no. 7, 1666–1671, 10.1128/jcm.34.7.1666-1671.1996. 8784566 PMC229091

[26] Murakami M. , Matsuba C. , Une Y. , Nomura Y. , and Fujitani H. , Development of Species-Specific PCR Techniques for the Detection of Tortoise Herpesvirus, Journal of Veterinary Diagnostic Investigation. (2001) 13, no. 6, 513–516, 10.1177/104063870101300610. 11724143

[27] Teifke J. , Löhr C. , Marschang R. , Osterrieder N. , and Posthaus H. , Detection of Chelonid Herpesvirus DNA by Nonradioactive in Situ Hybridization in Tissues From Tortoises Suffering From Stomatitis—Rhinitis Complex in Europe and North America, Veterinary Pathology. (2000) 37, no. 5, 377–385, 10.1354/vp.37-5-377. 11055860

[28] Marschang R. E. , Gleiser C. B. , Papp T. , Pfitzner A. J. , Böhm R. , and Roth B. N. , Comparison of 11 Herpesvirus Isolates From Tortoises Using Partial Sequences From Three Conserved Genes, Veterinary Microbiology. (2006) 117, no. 2–4, 258–266, 10.1016/j.vetmic.2006.06.009. 16857325

[29] Bender F. C. , Samanta M. , and Heldwein E. E. , et al.Antigenic and Mutational Analyses of Herpes Simplex Virus Glycoprotein B Reveal Four Functional Regions, Journal of Virology. (2007) 81, no. 8, 3827–3841, 10.1128/JVI.02710-06. 17267495 PMC1866100

[30] Coberley S. S. , Condit R. C. , Herbst L. H. , and Klein P. A. , Identification and Expression of Immunogenic Proteins of a Disease-Associated Marine Turtle Herpesvirus, Journal of Virology. (2002) 76, no. 20, 10553–10558, 10.1128/JVI.76.20.10553-10558.2002. 12239336 PMC136575

[31] Chiuppesi F. , Vannucci L. , and De Luca A. , et al.A Lentiviral Vector-Based, Herpes Simplex Virus 1 (HSV-1) Glycoprotein B Vaccine Affords Cross-Protection Against HSV-1 and HSV-2 Genital Infections, Journal of Virology. (2012) 86, no. 12, 6563–6574, 10.1128/JVI.00302-12. 22491465 PMC3393530

[32] Cunha C. W. , Baker K. N. , and O’Toole D. , et al.A Vaccine Targeting Ovine Herpesvirus 2 Glycoprotein B Protects Against Sheep-Associated Malignant Catarrhal Fever, Vaccines. (2022) 10, no. 12, 10.3390/vaccines10122156, 2156. 36560568 PMC9786699

[33] Humar A. , Kumar D. , Gilbert C. , and Boivin G. , Cytomegalovirus (CMV) Glycoprotein B Genotypes and Response to Antiviral Therapy, in Solid-Organ–Transplant Recipients With CMV Disease, The Journal of Infectious Diseases. (2003) 188, no. 4, 581–584, 10.1086/377002. 12898447

[34] Košovský J. , Vojvodová A. , Oravcová I. , Kúdelová M. , Matis J. , and Rajčáni J. , Herpes Simplex Virus 1 (HSV-1) Strain HSZP Glycoprotein B Gene: Comparison of Mutations Among Strains Differing in Virulence, Virus Genes. (2000) 20, no. 1, 27–33, 10.1023/A:1008104006007. 10766304

[35] Origgi F. C. , Tecilla M. , and Pilo P. , et al.A Genomic Approach to Unravel Host-Pathogen Interaction in Chelonians: The Example of Testudinid Herpesvirus 3, PLoS ONE. (2015) 10, no. 8, 10.1371/journal.pone.0134897, e0134897. 26244892 PMC4526542

[36] Kumar S. , Stecher G. , Li M. , Knyaz C. , Tamura K. , and Battistuzzi F. U. , MEGA X: Molecular Evolutionary Genetics Analysis Across Computing Platforms, Molecular Biology and Evolution. (2018) 35, no. 6, 1547–1549, 10.1093/molbev/msy096. 29722887 PMC5967553

[37] Cooper J. , Gschmeissner S. , and Bone R. , Herpes-Like Virus Particles in Necrotic Stomatitis of Tortoises, Veterinary Record. (1988) 123, no. 21, 544–544, 10.1136/vr.123.21.544. 3206806

[38] Drury S. E. N. , Gough R. E. , Kay A. V. , and McArthur S. D. J. , Detection and Isolation of a Herpesvirus From a Spur-Thighed Tortoise (*Testudo graeca*) in the UK, Veterinary Record. (1999) 145, no. 20, 586–588, 10.1136/vr.145.20.586. 10606021

[39] Hervás J. , Sánchez-Cordón P. J. , Cón De Lara F. C. H. A. , Carrasco L. , and Gómez-Villamandos J. C. , Hepatitis Associated With Herpes Viral Infection in the Tortoise (*Testudo horsfieldii*), Journal of Veterinary Medicine, Series B. (2002) 49, no. 2, 111–114, 10.1046/j.1439-0450.2002.00522.x. 12002420

[40] Adler B. , Sattler C. , and Adler H. , Herpesviruses and Their Host Cells: A Successful Liaison, Trends in Microbiology. (2017) 25, no. 3, 229–241, 10.1016/j.tim.2016.11.009. 27956142

[41] Gómez-Villamandos J. , Mozos E. , Sierra M. , Fernandez A. , and Diaz F. , Mortality in Psittacine Birds Resembling Pacheco’s Disease in Spain, Avian Pathology. (1991) 20, no. 3, 541–547, 10.1080/03079459108418791. 18680049

[42] Knipe D. , Howley P. , and Griffin D. , et al.Fields Virology, 2013, 1, 6 edition, Lippincott Williams & Wilkins, 2479–2500, Chapter 66.

[43] O’Donnell C. D. and Shukla D. , The Importance of Heparan Sulfate in Herpesvirus Infection, Virologica Sinica. (2008) 23, no. 6, 383–393, 10.1007/s12250-008-2992-1. 19956628 PMC2778322

[44] Kolesnik E. , Obiegala A. , and Marschang R. E. , Detection of Mycoplasma Spp., Herpesviruses, Topiviruses, and Ferlaviruses in Samples From Chelonians in Europe, Journal of Veterinary Diagnostic Investigation. (2017) 29, no. 6, 820–832, 10.1177/1040638717722387. 28754074

[45] Marschang R. , Gravendyck M. , and Kaleta E. , Herpesviruses in Tortoises: Investigations Into Virus Isolation and the Treatment of Viral Stomatitis in *Testudo hermanni* and *T. graeca* , Journal of Veterinary Medicine, Series B. (1997) 44, no. 1–10, 385–394, 10.1111/j.1439-0450.1997.tb00989.x. 9323927

[46] Grinde B. , Herpesviruses: Latency and Reactivation–Viral Strategies and Host Response, Journal of Oral Microbiology. (2013) 5, no. 1, 10.3402/jom.v5i0.22766, 22766. PMC3809354 24167660

[47] Worrall G. , Herpes Labialis, BMJ Clinical Evidence. (2009) 2009. PMC2907798 21726482

[48] Sawtell N. and Thompson R. , Rapid In Vivo Reactivation of Herpes Simplex Virus in Latently Infected Murine Ganglionic Neurons After Transient Hyperthermia, Journal of Virology. (1992) 66, no. 4, 2150–2156, 10.1128/jvi.66.4.2150-2156.1992. 1312625 PMC289007

[49] Jacobson E. R. , Brown M. B. , and Wendland L. D. , et al.Mycoplasmosis and Upper Respiratory Tract Disease of Tortoises: A Review and Update, The Veterinary Journal. (2014) 201, no. 3, 257–264, 10.1016/j.tvjl.2014.05.039. 24951264

